# Sleep Apnea and Substance Use Disorders Associated with Co-Occurrence of Anxiety Disorder and Depression among U.S. Adults: Findings from the NSDUH 2008–2014

**DOI:** 10.3390/brainsci13040661

**Published:** 2023-04-14

**Authors:** Chun Xu, Priscila Acevedo, Liang Wang, Nianyang Wang, Kaysie Ozuna, Saima Shafique, Annu Karithara, Victoria Padilla, Chunxiang Mao, Xin Xie, Kesheng Wang

**Affiliations:** 1 Department of Health and Biomedical Sciences, College of Health Professions, University of Texas Rio Grande Valley, Brownsville, TX 78520, USA; kaysie.ozuna01@utrgv.edu (K.O.); annu.karithara01@utrgv.edu (A.K.); victoria.padilla01@utrgv.edu (V.P.); frankmao2007@gmail.com (C.M.); 2 School of Biomedical Informatics, University of Texas Health Science Center at Houston, Houston, TX 77030, USA; priscila.acevedo@uth.tmc.edu; 3 Department of Public Health, Robbins College of Health and Human Sciences, Baylor University, Waco, TX 76798, USA; liang_wang1@baylor.edu; 4 Department of Health Policy and Management, School of Public Health, University of Maryland, College Park, MD 20742, USA; nwang123@umd.edu; 5 School of Nursing, Health Sciences Center, West Virginia University, Morgantown, WV 26506, USA; ss0093@mix.wvu.edu; 6 Department of Economics and Finance, College of Business and Technology, East Tennessee State University, Johnson City, TN 37614, USA; xiex01@etsu.edu

**Keywords:** anxiety disorder, depression, co-occurrence, sleep apnea, substance use disorders, multinomial logistic regression

## Abstract

Few studies have focused on sleep apnea and substance use disorders with co-occurrence of anxiety disorder and depression. This study included a total of 270,227 adults, 9268 with co-occurrence of anxiety disorder and depression in the past year, from the combined 2008–2014 National Survey on Drug Use and Health (NSDUH) data, which are the latest datasets with measures of anxiety disorder and sleep apnea. Weighted multinomial logistic regression analyses were used to estimate the associations between anxiety disorder and depression and their co-occurrence. Comorbidity was highly prevalent: 40.4% of those with depression also met the criteria for anxiety disorder, whereas 51.8% of those with anxiety disorder also met the criteria for depression. The prevalences of anxiety only and co-occurrence increased from 2008 to 2014. The prevalences of anxiety disorder only, depression only, and co-occurrence of anxiety disorder and depression in individuals with sleep apnea were 4.4%, 12.9%, and 12.2%, respectively, and the prevalences in substance use disorders were 6.4%, 9.4%, and 10.7%, respectively. The results showed that sleep apnea, substance use disorders, and nicotine dependence were significantly associated with increased odds of anxiety disorder, depression, and co-occurrence (all *p* values < 0.0001). Furthermore, several chronic diseases (asthma, bronchitis, hypertension, and heart disease) were associated with the co-occurrence of anxiety disorder and depression. These findings suggest clinicians and other healthcare providers consider screening for depression and anxiety with sleep apnea and substance use disorders for improved therapeutic outcomes.

## 1. Introduction

Anxiety and depression are the two most reported mental health disorders among the general population and are often comorbid [1,2,3]. Anxiety disorder is a highly prevalent and disabling mental health condition where a temporary worry or fear is heightened to a point where structural and functional changes in the brain are observed [4]. Depression is a widespread mental healthcare problem and a common cause of disability. Previous studies have shown that low self-esteem, a family history of depression, childhood sexual abuse, early traumatic experiences, years of education, and disturbed family environments are risk factors for anxiety and depression [5,6]. In a recent review study, female sex, financial difficulties, low socioeconomic status, a lack of social support, a history of eating disorders, stress, a low education level, alcohol and smoking, and chronic illnesses were associated with depression [7].

Sleep apnea is commonly found to be comorbid with mental disorders such as anxiety, post-traumatic stress disorder, schizophrenia, depression [8,9], heart failure, and chronic opiate use [10]. A recent study reported that sleep apnea was associated with mental disorders, including anxiety and depression [11], while another study found that sleep apnea patients had higher levels of depression and anxiety [12]. Additionally, one more recent study found that depressive and anxious symptoms were prevalent in sleep apnea; however, sleep apnea severity did not contribute to depression and to anxiety [13].

Increasing evidence suggests that substance use disorders (SUDs) have been also associated with anxiety [14,15] and depression [16,17,18]. For example, alcohol, cannabis, and tobacco use in people with anxiety disorders was commonly reported, and tobacco use was significantly associated with anxiety symptoms [14]. Interestingly, e-cigarettes were also associated with various mental health and drug use problems [15]. Furthermore, alcohol use disorder [19], marijuana use [20], and cannabis use disorder [21] often occur with depression and anxiety disorders. In addition, SUDs are commonly comorbid with anxiety and depressive disorders and are associated with poor treatment outcomes [22,23,24].

However, few studies have focused on sleep problems or sleep apnea and SUDs with the co-occurrence of anxiety disorder and depression [25,26,27]. The present study aimed to examine the prevalence of anxiety disorder only, depression only, and their co-occurrence and detect the associations of sleep apnea and SUDs with anxiety disorder only, depression only, and their co-occurrence among adults in the United States (U.S.) using the 2008–2014 National Survey on Drug Use and Health (NSDUH), which contains the latest datasets with measures of anxiety disorder and sleep apnea. Previous studies have examined sleep apnea [8] and anxiety disorder/depression [28,29,30,31] using NSDUH data. However, no study has used NSDUH data to examine sleep apnea and SUDs with the co-occurrence of anxiety disorder and depression.

## 2. Materials and Methods

### 2.1. Study Population

This is a secondary data analysis of cross-sectional publicly available survey data. The combined 2008–2014 NSDUH data sample size in the present study was extracted from the 2008–2014 National Survey on Drug Use and Health (NSDUH). The Substance Abuse and Mental Health Services Administration of the U.S. Department of Health and Human Services conducts the NSDUH. The NSDUH surveys civilian non-institutionalized individuals (≥12 years old) in the U.S., providing annual population estimates on substance use and health outcomes. The NSDUH measures the prevalence and correlations of drug use in the U.S. quarterly and annually. The survey contains data on illicit drugs, alcohol, and tobacco and the nonmedical use of prescription drugs. The survey includes questions from the Diagnostic and Statistical Manual (DSM) about mental disorders, substance abuse treatment history, and the perceived need for treatment. In addition, the survey asks for information on whether the person had sleep apnea, anxiety, or a major depressive episode in the past year. The additional background information of the respondents includes sex, race/ethnicity, age, marital status, educational level, job status, veteran status, and current household composition. The combined 2008–2014 NSDUH data sample size is 391,753 [32]. The current analysis was restricted to participants ages 18 and older, resulting in a total sample size of 270,227. There was an Institutional Review Board exemption for the current study due to secondary data analysis.

### 2.2. Measurements

#### 2.2.1. Outcome Measure

Anxiety disorder and depression in the past year were defined as “yes” if they responded “yes” to the question asking whether either a doctor or another medical professional has ever told them that they had anxiety or depression in the past year. The outcome variable was further categorized into anxiety disorder only, depression only, and co-occurrence of anxiety disorder and depression.

#### 2.2.2. Social-Demographic Factors

Sex was self-reported as male or female. Age was classified into four categories: 18–25, 26–49, 50–64, and 65+ years. Race/ethnicity was categorized as White, African American (AA), Asian, Hispanic, and others. There were three categories of marriage status: married, widowed/divorced/separated, and never been married. The four annual income categories were USD < 20,000, USD 20,000–49,999, USD 50,000–74,999, and USD 75,000 or more.

#### 2.2.3. Chronic Diseases

Sleep apnea, bronchitis, diabetes, and heart disease in the past year were dichotomized into “yes” or “no.” Participants were considered to have one of the above conditions if they responded “yes” to the question asking whether a doctor or another medical professional has ever told them about having a condition in the past year. Subjects were considered to have had current asthma if they responded “yes” to the questions “Ever been told you had asthma” and “Still have asthma.” Hypertension was defined as “yes” if they responded “yes” to the question asking whether a doctor or another medical professional has ever told them that they had high blood pressure in the past year.

#### 2.2.4. Substance Use Disorders in the Past Year

Nicotine dependence was defined as the average score over 17 questions about five aspects of the Nicotine Dependence Syndrome Scale (NDSS). These average scores were computed for respondents answering all the questions [33]. Illicit drug or alcohol abuse or dependence in the past year was defined as being dependent on any illicit drug or alcohol in the past year or illicit drug or alcohol abuse in the past year based on the DSM-IV [34]. Illicit drug abuse was defined as abusing any of the following substances: marijuana, hallucinogens, inhalants, tranquilizers, cocaine, heroin, pain relievers, stimulants, or sedatives. Illicit drug dependence was classified as being dependent on any of these following substances: marijuana, hallucinogens, inhalants, tranquilizers, cocaine, heroin, pain relievers, stimulants, or sedatives. We used SUDs as a combined variable of abuse or dependence on illicit drugs and/or alcohol [35]. Illicit drug or alcohol dependence or abuse was referred to as an SUD, illicit drug dependence or abuse was referred to as an illicit drug use disorder (IDUD), and alcohol dependence or abuse was referred to as an alcohol use disorder (AUD).

### 2.3. Statistical Analysis

The SAS PROC SURVEYFREQ was used to weigh and estimate the population proportions of anxiety disorder only, depression only, and their co-occurrence. The overall prevalence and the prevalences of potential factors were estimated. The Chi-square test was used to compare the prevalence across age groups, sex, race, and other factors.

SAS PROC SURVEYLOGISTIC was used to estimate the odds ratios (ORs) with 95% confidence intervals (Cis) for the associations of potential factors for anxiety disorder only, depression only, and their co-occurrence. Two models were conducted. In model 1, a bivariate multinomial logistic regression was used to examine the role of each potential risk factor in anxiety disorder only, depression only, and co-occurrence of anxiety disorder and depression. In model 2, a multivariable multinomial logistic regression was used to adjust for all the potential risk factors of anxiety disorder only, depression only, and co-occurrence of anxiety disorder and depression. All analyses were performed using SAS statistical software, version 9.4 (SAS Institute, Cary, NC, USA).

## 3. Results

### 3.1. Overall Prevalence of Anxiety and Depression and Comorbidity in the Population

The overall prevalences of anxiety disorder and depression in the population were 5.9% and 7.5%, respectively (Table 1). Comorbidity was highly prevalent: 40.4% of those with depression also met the criteria for anxiety disorder, whereas 51.8% of those with anxiety disorder also met the criteria for depression.

### 3.2. Prevalence of Anxiety Only, Depression Only, and Their Co-Occurrence by Year

The overall prevalences of anxiety only, depression only, and their co-occurrence were 2.8%, 4.5%, and 3.0%, respectively (Table 2). The prevalence from 2008 to 2014 can also be reported followed by increasing trends over the years. The prevalence of anxiety only and the co-occurrence increased from 2008 to 2014.

### 3.3. Prevalence of Anxiety Only, Depression Only, and Their Co-Occurrence across Potential Factors

Prevalences of anxiety only, depression only, and their co-occurrence were higher in females than in males (3.6% for females vs. 2.0% for males, 5.9% for females vs. 3.0% for males, and 4.2% for females vs. 1.8% for males, respectively) (Table 3). Overall, young age groups tended to suffer from depression, anxiety, and co-occurrence more than the oldest age group (≥65 years). Regarding the prevalence based on race, Whites had the highest prevalence of anxiety, depression, and co-occurrence compared with other ethnic groups. We also found that widowed, divorced, or separated people had the highest prevalence of anxiety compared with married people. The participants having a low income had an increased prevalence of depression, anxiety, and co-occurrence compared with those with a high income.

**Figure 1:**
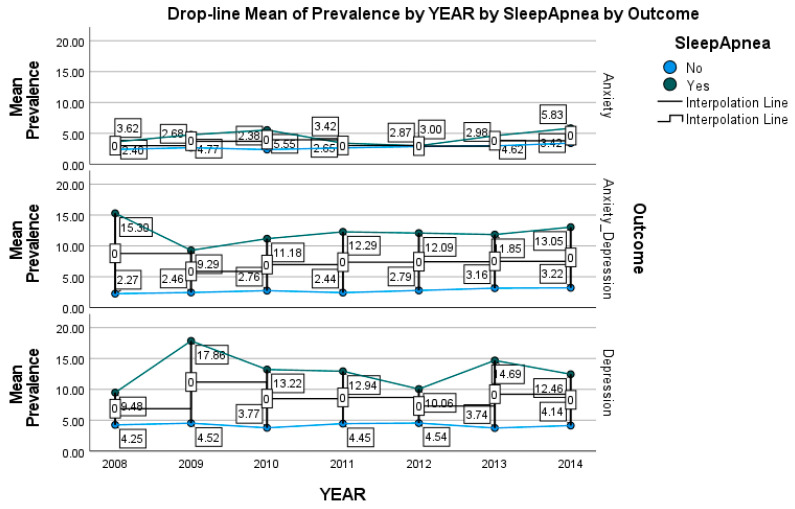
Prevalence of anxiety only, depression only, and their co-occurrence by year and sleep apnea.

The prevalence of anxiety only, depression only, and co-occurrence was higher in those with asthma, hypertension, and obesity when compared with the subjects without those health conditions (Table 3). Furthermore, the prevalences of anxiety disorder only, depression only, and co-occurrence of anxiety disorder and depression in individuals with SUDs were 6.4%, 9.4%, and 10.7%, respectively, and prevalences in individuals with nicotine dependence were 4.7%, 6.5%, and 6.6%, respectively. Among individuals with sleep apnea, the prevalences of anxiety disorder, depression, and co-occurrence were 4.4%, 12.9%, and 12.2%, respectively (Table 3). Figure 1 illustrates the prevalence of anxiety disorder, depression, and co-occurrence by year and sleep apnea status. Especially, the prevalence of anxiety increased from 3.62% in 2008 to 5.83% in 2014.

### 3.4. Multivariable Logistic Regression Analyses

Table 4 depicts the results of the multinomial logistic regression analyses adjusted for covariates. Females had higher odds in anxiety, depression, or both conditions, whereas seniors had lower odds in anxiety, depression, or both conditions. Compared with the married category, the never married and the divorced, widowed, or separated categories were significantly associated with higher odds of anxiety, depression, and the comorbidity of both. In addition, subjects who were AA, Asian, and Hispanic were less likely to develop anxiety, depression, and both conditions (all *p*-values < 0.0001) than the White population. A higher annual income was significantly associated with lower odds of anxiety, depression, and both conditions.

Asthma was associated with anxiety, depression, and both conditions (OR = 1.22, 95% CI = 1.06–1.41; OR = 1.28, 95% CI = 1.15–1.42; and OR = 1.87, 95% CI = 1.64–2.13, respectively). Bronchitis, hypertension, and heart disease were associated with increased odds of depression only and depression and anxiety comorbidity. Sleep apnea was significantly associated with increased odds of anxiety disorder, depression, and co-occurrence (OR = 2.44, 95% CI = 2.03–2.94; OR = 3.73, 95% CI = 3.27–4.25; and OR = 5.39, 95% CI = 4.67–6.22, respectively). Nicotine dependence was associated with anxiety, depression, and both conditions (OR = 1.74, 95% CI= 1.60–1.89; OR = 1.44, 95% CI = 1.33–1.56; and OR = 2.07, 95% CI= 1.88–2.27, respectively). SUDs were associated with anxiety, depression, and their co-occurrence (OR = 2.51, 95% CI= 2.12–2.98; OR = 2.99, 95% CI = 2.58–3.47; and OR = 3.83, 95% CI= 3.17–4.62, respectively).

## 4. Discussion

The findings from a large population-based and representative sample of the U.S. population reported that sleep apnea and SUDs were positively associated with the co-occurrence of anxiety disorder and depression. The present study also provided the overall prevalence of anxiety disorder, depression, and their co-occurrence in the U.S. population over seven years. Furthermore, these conditions were more prevalent among those with sleep apnea and SUDs, including alcohol and/or illicit drug use, than those without SUDs.

Depression and co-occurrence both are common conditions and prevalence varies with demographic factors, which is consistent with previous studies [36,37]. Furthermore, 65 years and older people have the lowest prevalence of both disorders than younger participants between 18–25 years. This is similar to a previous study which showed that the first depressive episode is more likely to happen between adolescence and mid-40s, with its peak prevalence between 20 to 40 years and 50 to 70 years [38]. Another study also reported that the prevalence of depression to be the lowest in adults 65+ years old compared with younger age groups [39].

The present study found that the White population had the highest prevalence of anxiety, depression, and both conditions compared with other ethnic groups. A recent study also supports our findings, where the authors reported that in the U.S., Black and Hispanic minority participants seemed to have better mental health than non-Hispanic Whites [40]. Widowed, separated, or divorced individuals had a higher prevalence of depression and co-occurrence of anxiety and depression, which was similar to previous studies among women [41]. A recent study found that marital loss was associated with increased depressive symptoms, whereas marital gain was associated with decreased depressive symptoms [42]. A higher prevalence of anxiety, depression, and both conditions was noted among those with lower socio-economic status (USD < 49,999), which was in agreement with a study that found that having a low income (USD < 40,000) increased the odds of mental disorders, including depressive and anxiety disorders [43].

A person’s overall health status may play a role in developing depression, anxiety, and the comorbidity of both, similar to a recent meta-analysis [44]. Poor health conditions may increase the risk of mental disorders. Having asthma increases the odds of developing anxiety, depression, and both conditions, as documented by others [45]. Furthermore, those with sleep apnea have a twofold increased risk of anxiety and the comorbidity of both conditions compared with those without sleep apnea. This finding supports previous studies including one study conducted in Canada, where the patients with obstructive sleep apnea (OSA) had higher levels of depression and anxiety in a small sample size of 102 participants [12]. A meta-analysis based on 73 articles showed significant associations between OSA, anxiety, and depression, suggesting that the early diagnosis and effective treatment of OSA can improve mental disorders such as depression or anxiety [11]. Moreover, a study using earlier data from NSDUH showed that sleep apnea increased the odds of anxiety, depression, and suicidal ideation [8]. However, this study did not examine sleep apnea with the co-occurrence of anxiety and depression. A review publication described that anxiety in sleep apnea negatively impacts the quality of life and bidirectional relationships [46]. A recent study showed that shorter sleep duration and worse subjective sleep quality, as well as depression, were associated with increased odds of suicidal ideation [47]. The association between sleep apnea, anxiety, and depression based on the findings of the current and previous studies indicates the importance of an early diagnosis and personalized treatment of sleep apnea to improve mental conditions’ compliance to therapy. However, these conditions probably share a bidirectional relationship. Thus, additional research is needed to understand the relationship between sleep apnea, anxiety, and depression. It is essential to determine the underlying mechanisms responsible for the co-occurrence of sleep apnea, anxiety, and depression and introduce effective preventive measures to improve health outcomes. These findings have implications for sleep clinicians, other health practitioners, and researchers to focus on the risk of comorbid sleep apnea with other mental disorders in their communities.

Another key finding of the current study is that there was a higher prevalence of mental disorders in those with SUDs and nicotine dependence. Furthermore, multinomial logistic regression analyses supported that SUDs and nicotine dependence were strongly associated with increased odds of anxiety, depression, and their co-occurrence. One previous systematic review and meta-analysis has confirmed that there is a strong association between SUDs, mood disorders, and anxiety disorders [48]. Several studies have shown that substance use or SUDs were associated with anxiety [14,15] and depression [7,16,17,18]. One recent study found that nicotine dependence plays a vital role in an individual’s anxiety and depression levels [49]. Another study showed that there was a correlation between nicotine dependence and depression [50]. One recent review study also supported that anxiety and depression are often comorbid with tobacco dependence, partly reflecting attempts to self-medicate symptoms with nicotine [51]. However, substance use and its relationship with mental health conditions and the underlying factors vary among different fractions of populations. For example, alcohol and cannabis use were not found to be associated with anxiety symptomatology [14]. Another study found an association of poly-substance use with depression but not with anxiety among Canadian adolescents [52]. Furthermore, one study showed that the use of e-cigarettes is common among university students and is associated with various mental health and drug use problems [15]. In addition, there were inconsistent results regarding whether smoking caused anxiety and/or depression or vice versa [53]. The present study added that nicotine dependence and SUDs were associated with anxiety only, depression only, and their co-occurrence among U.S. adults. However, depressive or anxiety disorders may be risk factors for nicotine dependence and SUDs. For example, adolescents with depression and anxiety symptoms are at increased risk for alcohol use disorders into young adulthood [54], whereas long-term concurrent users of cigarettes, alcohol, and marijuana were more likely to show generalized anxiety and depression in adulthood compared with occasional users of alcohol [55]. One recent study provided evidence for a positive association between mental health problems and substance experimentation in childhood [56]. Another study found that increasing severity of depression risk is associated with increasingly risky substance use, whereas among those with depression risk, comorbid severe anxiety risk further increases risky use [57]. One recent longitudinal study found that there are longitudinal reciprocal associations between depression and SUD in young and middle adulthood over a period of 30 years, whereas anxiety negatively predicted SUD in young adulthood, while SUD did not predict subsequent anxiety [58]. However, the mechanisms about the relationships among depression, anxiety, and substance use are not clear. One family study supports the co-occurrence of depression, anxiety, obesity, and substance use within families and provides estimates of genetic sharing underlying the familial co-occurrence [59]. Another study found that several miRNAs were associated with anxiety and depression in SUD patients and suggested that four DE-miRNAs may be potential biomarkers for the diagnosis and treatment of anxiety and depression in SUDs [24].

### Strengths and Limitations

Our study has several strengths. First, this study combined 2008–2014 NSDUH data to create a large sample size in examining the prevalence of the co-occurrence of anxiety disorder and depression. This large sample size using a national survey allowed us to use diverse demographic variables and adjust for various factors, including race/ethnicity and chronic diseases. Second, to the best of our knowledge, our present study is the first attempt to examine the association of sleep apnea and SUDs with the co-occurrence of anxiety and depression. We used the weighted multinomial logistic regression analyses to estimate the associations of sleep apnea, nicotine dependence, SUDs, and other factors with anxiety disorder only, depression only, and their co-occurrence.

Despite the strengths, the study has a few limitations that must be acknowledged. First, the cross-sectional study design could not draw causal inferences; a longitudinal study is recommended to prove the association. Second, self-reported data on substance use and health conditions may suffer from social desirability and recall bias. Self-reported sleep apnea was too broad to be a specific type and heterogenous population since there are three types of sleep apnea: obstructive, central, and mixed. Future studies focusing on specific types are suggested. A previous study has shown that substance use reported by NSDUH respondents is generally valid [60], whereas the NSDUH data were collected by self-reported data on substance use and health conditions, making responses prone to social desirability bias and recall bias. Additionally, the current study only used NSDUH data till 2014 because since 2015, the measures of anxiety disorder and sleep apnea were not available in the NSDUH data.

## 5. Conclusions

In summary, anxiety, depression, and their co-occurrence were common among females, younger age groups, non-Hispanic Whites, people with separated/divorced/never-married marital status, and low-income groups. Most importantly, we identified the strongest associations of sleep apnea, nicotine dependence, and SUDs with anxiety, depression, and their co-occurrence in a large representative sample. Furthermore, chronic conditions such as asthma may be associated with anxiety, depression, and their co-occurrence. Thus, clinicians and other healthcare providers may consider screening for depression and anxiety alongside sleep apnea to improve therapeutic outcomes. In addition, strategies for promoting healthy behaviors should consider critical factors such as race/ethnicity, age, sex, and chronic conditions on mental health among young adults.

## Figures and Tables

**Table 1:** Prevalence of anxiety, depression, and comorbidity (%).

Variable	Total	Anxiety	Prevalence (%) (95% CI)	Total	Depression	Prevalence (%) (95% CI)	*p*-Value
Anxiety							
No				248,866	11,561	4.8 (4.6–4.9)	<0.0001
Yes				9268	8142	51.8 (50.6–53.0)	
Depression							
No	245,447	8142	3.1 (3.0–3.2)				<0.0001
Yes	20,829	9268	40.4 (39.3–41.5)				
Overall	270,227	17,410	5.9 (5.7–6.0)	270,227	20,829	7.5 (7.4–7.7)	

Abbreviations: 95% CI = 95% confidence interval; comorbidity refers to individuals with both anxiety and depression in anxiety or depression individuals; *p*-value is based on the χ^2^ test.

**Table 2:** Prevalence of anxiety only, depression only, and their co-occurrence by year (%).

Variable	Total	Anxiety Only	Prevalence (%) (95% CI)	Depression Only	Prevalence (%) (95% CI)	Co-Occurrence	Prevalence (%) (95% CI)	^a^ *p*-Value
Year								
2008	36,910	939	2.4 (2.1–2.7)	1630	4.4 (4.0–4.8)	1018	2.6 (2.4–2.9)	<0.0001
2009	37,182	1029	2.7 (2.5–3.0)	1662	4.9 (4.6–5.2)	1135	2.6 (2.4–2.9)	
2010	38,343	1062	2.5 (2.2–2.7)	1636	4.0 (3.7–4.4)	1288	3.0 (2.7–3.2)	
2011	38,613	1099	2.7 (2.4–2.9)	1717	4.7 (4.4–5.1)	1250	2.8 (2.5–3.0)	
2012	37,331	1190	2.9 (2.6–3.2)	1683	4.7 (4.3–5.1)	1343	3.1 (2.9–3.4)	
2013	36,834	1285	3.0 (2.7–3.3)	1508	4.2 (3.8–4.5)	1489	3.5 (3.2–3.8)	
2014	41,063	1538	3.5 (3.3–3.7)	1725	4.5 (4.2–4.7)	1745	3.6 (3.4–3.9)	
^b^ *p*-Value			<0.0001		0.0047		<0.0001	
Overall	270,227	8142	2.8 (2.7–2.9)	11,561	4.5 (4.4–4.6)	9268	3.0 (2.9–3.1)	

Abbreviations: CI = confidence interval; co-occurrence refers to individuals with both anxiety and depression in the population; ^a^ *p*-value is based on the χ^2^ test for the year with disease status; ^b^ *p*-value is based on the χ^2^ test for the year with anxiety only, depression only, and co-occurrence.

**Table 3:** Prevalence of anxiety only, depression only, and their co-occurrence (%).

Variable	Total	Anxiety Only	Prevalence (%) (95% CI)	Depression Only	Prevalence (%) (95% CI)	Co-Occurrence	Prevalence (%) (95% CI)	*p*-Value
Sex								
Male	123,623	2606	2.0 (1.9–2.1)	3471	3.0 (2.9–3.2)	2377	1.8 (1.7–1.9)	<0.0001
Female	142,653	5536	3.6 (3.5–3.8)	8090	5.9 (5.6–6.1)	6891	4.2 (4.0–4.3)	
Age group								
18–25 years	123,552	3657	3.0 (2.8–3.1)	4591	3.4 (3.3–3.5)	4067	3.1 (3.0–3.2)	<0.0001
26–49 years	98,308	3389	3.2 (3.1–3.4)	4649	4.4 (4.2–4.6)	3968	3.5 (3.4–3.7)	
50–64 years	27,400	732	2.6 (2.3–2.8)	1714	5.9 (5.5–6.3)	1018	3.4 (3.1–3.7)	
65+ years	17,016	364	2.2 (1.9–2.4)	607	3.6 (3.2–4.0)	215	1.3 (1.1–1.5)	
Race								
White	167,953	6332	3.4 (3.3–3.5)	8507	5.2 (5.0–5.3)	7442	3.7 (3.6–3.8)	<0.0001
AA	32,974	508	1.6 (1.4–1.8)	936	3.0 (2.7–3.3)	435	1.3 (1.1–1.5)	
Asian	10,525	109	1.0 (0.6–1.3)	176	1.8 (1.3–2.2)	101	1.1 (0.7–1.5)	
Hispanic	41,981	804	1.8 (1.6–2.0)	1319	3.3 (3.0–3.7)	785	1.8 (1.6–2.1)	
Other	12,843	389	2.7 (2.1–3.3)	623	5.1 (4.1–6.2)	505	3.9 (3.3–4.6)	
Marital status								
Married	97,095	2791	2.5 (2.4–2.7)	4197	4.1 (3.9–4.3)	2807	2.4 (2.3–2.5)	<0.0001
Widowed/divorced/separated	32,387	1204	3.2 (2.9–3.5)	2149	6.2 (5.8–6.6)	1825	4.3 (4.1–4.7)	
Never married	136,794	4147	3.2 (3.0–3.4)	5215	3.9 (3.8–4.1)	4636	3.4 (3.2–3.5)	
Income								
Less than USD 20,000	66,665	2294	3.4 (3.1–3.7)	3329	5.6 (5.3–6.0)	3089	4.7 (4.5–4.9)	<0.0001
USD 20,000–49,999	90,208	2626	2.8 (2.6–3.0)	3904	4.6 (4.4–4.8)	3057	3.1 (2.9–3.3)	
USD 50,000–74,999	42,423	1220	2.5 (2.3–2.7)	1737	4.4 (4.1–4.8)	1284	2.8 (2.6–3.0)	
USD 75,000 or more	66,980	2002	2.7 (2.6–2.9)	2591	3.8 (3.6–3.9)	1838	2.2 (2.0–2.3)	
Sleep apnea								
No	260,700	7843	2.8 (2.7–2.9)	10,791	4.2 (4.1–4.3)	8396	2.7 (2.6–2.8)	<0.0001
Yes	5550	299	4.4 (3.7–5.2)	770	12.9 (11.6–14.3)	872	12.2 (10.9–13.5)	
IDUD and AUD								
No	260,910	7818	2.8 (2.7–2.9)	11,138	4.4 (4.3–4.6)	8724	3.0 (2.9–3.1)	<0.0001
Yes	5366	324	6.4 (5.5–7.4)	423	9.4 (8.2–10.7)	544	10.7 (9.0–12.4)	
Nicotine dependence								
No	224,510	6070	2.5 (2.4–2.6)	8815	4.2 (4.0–4.3)	6324	2.5 (2.4–2.6)	<0.0001
Yes	41,766	2072	4.7 (4.4–5.1)	2746	6.5 (6.1–6.9)	2944	6.6 (6.2–6.9)	
Asthma								
No	248,382	7461	2.8 (2.7–2.9)	10,480	4.4 (4.2–4.5)	7880	2.8 (2.7–2.9)	<0.0001
Yes	17,772	681	3.6 (3.1–4.1)	1081	6.6 (6.0–7.2)	1388	7.4 (6.6–8.1)	
Bronchitis								
No	256,713	7755	2.8 (2.7–2.9)	10,911	4..4 (4.2–4.5)	8446	2.8 (2.7–2.9)	<0.0001
Yes	9510	387	3.8 (3.2–4.4)	650	7.4 (6.7–8.1)	822	7.9 (7.1–8.7)	
Diabetes								
No	255,617	7917	2.9 (2.8–3.0)	10,870	4.4 (4.2–4.5)	8703	3.0 (2.9–3.1)	<0.0001
Yes	10,606	225	1.9 (1.6–2.2)	691	6.0 (5.3–6.6)	565	4.1 (3.5–4.7)	
Hypertension								
No	237,705	7302	2.9 (5.8–3.0)	9684	4.1 (3.9–4.2)	7723	2.7 (2.7–2.8)	<0.0001
Yes	28,471	840	2.4 (2.2–2.7)	1877	6.3 (5.9–6.7)	1545	4.3 (4.0–4.6)	
Heart disease								
No	261,172	8002	4.2 (4.0–4.5)	11,227	4.3 (4.1–4.5)	8938	2.1 (2.0–2.3)	<0.0001
Yes	5082	140	2.8 (2.7–2.9)	334	6.4 (5.4–7.4)	330	4.9 (4.1–5.7)	

Abbreviations: AA refers to African American; IDUD refers to illicit drug use disorder; AUD refers to alcohol use disorder. CI refers to confidence interval; co-occurrence presents both anxiety and depression; *p*-value is based on the χ^2^ test.

**Table 4:** Multinomial logistic regression analyses of potential factors with anxiety only, depression only, and their co-occurrence.

Variable	Anxiety Only OR (95% CI)	*p*-Value	Depression Only OR (95% CI)	*p*-Value	Co-Occurrence OR (95% CI)	*p*-Value
Year (ref = 2008)						
2009	1.14 (0.98–1.33)	0.0886	1.12 (1.01–1.25)	0.0313	1.02 (0.89–1.17)	0.7455
2010	1.02 (0.87–1.20)	0.7665	0.91 (0.80–1.04)	0.1814	1.14 (1.01–1.30)	0.0400
2011	1.13 (0.96–1.33)	0.1361	1.08 (0.97–1.21)	0.1716	1.06 (0.91–1.25)	0.4341
2012	1.24 (1.04–1.46)	0.0152	1.10 (0.96–1.26)	0.1565	1.26 (1.11–1.43)	0.0006
2013	1.32 (1.14–1.54)	0.0003	0.97 (0.85–1.11)	0.6559	1.42 (1.25–1.61)	<0.0001
2014	1.56 (1.35–1.79)	<0.0001	1.06 (0.95–1.18)	0.2803	1.50 (1.33–1.69)	<0.0001
Sex (ref = male)	2.13 (1.99–2.29)	<0.0001	2.26 (2.11–2.41)	<0.0001	2.83 (2.61–3.06)	<0.0001
Age group (ref = 18–34)						
35–49	1.30 (1.20–1.40)	<0.0001	1.36(1.26–1.47)	<0.0001	1.29 (1.19–1.40)	<0.0001
50–64	0.98 (0.85–1.14)	0.8308	1.50 (1.34–1.68)	<0.0001	0.91 (0.80–1.03)	0.1172
65+	0.71 (0.61–0.84)	<0.0001	0.68 (0.59–0.80)	<0.0001	0.23 (0.19–0.29)	<0.0001
Race (ref = White)						
AA	0.36 (0.31–0.42)	<0.0001	0.42 (0.38–0.47)	<0.0001	0.20(0.18–0.39)	<0.0001
Hispanic	0.47 (0.42–0.54)	<0.0001	0.61 (0.55–0.68)	<0.0001	0.43 (0.38–0.49)	<0.0001
Asian	0.69 (0.55–0.85)	<0.0001	0.86 (0.69–1.07)	<0.0001	0.77 (0.63–0.95)	<0.0001
Marital status (ref = married)						
Widowed/divorced/separated	1.20 (1.05–1.37)	0.0008	1.33 (1.23–1.44)	<0.0001	1.51 (1.37–1.68)	<0.0001
Never married	1.28 (1.15–1.43)	<0.0001	1.12 (1.03–1.22)	0.0064	1.35 (1.23–1.49)	<0.0001
Income (ref = less than USD 20,000)						
USD 20,000–49,999	0.81 (0.73–0.89)	<0.0001	0.81 (0.73–0.87)	<0.0001	0.71 (0.65–0.77)	<0.0001
USD 50,000–74,999	0.70 (0.61–0.80)	<0.0001	0.74 (0.66–0.84)	<0.0001	0.63 (0.55–0.71)	<0.0001
USD 75,000 or more	0.77 (0.68–0.87)	<0.0001	0.63 (0.57–0.70)	<0.0001	0.50 (0.44–0.57)	<0.0001
Sleep apnea (ref = no)	2.44 (2.03–2.94)	<0.0001	3.73 (3.27–4.25)	<0.0001	5.39 (4.67–6.22)	<0.0001
IDUD and AUD (ref = no)	2.51 (2.12–2.98)	<0.0001	2.99 (2.58–3.47)	<0.0001	3.83 (3.17–4.62)	<0.0001
Nicotine dependence (ref = no)	1.74 (1.60–1.89)	<0.0001	1.44 (1.33–1.56)	<0.0001	2.07 (1.88–2.27)	<0.0001
Asthma (ref = no)	1.22 (1.06–1.41)	0.0079	1.28 (1.15–1.42)	<0.0001	1.87 (1.64–2.13)	<0.0001
Bronchitis (ref = no)	1.15 (0.97–1.36)	0.1134	1.27 (1.13–1.43)	<0.0001	1.68 (1.48–1.91)	<0.0001
Diabetes (ref = no)	0.77 (0.64–0.92)	0.0044	1.15 (1.00–1.32)	0.0448	1.19 (0.99–1.42)	0.0667
Hypertension (ref = no)	0.99 (0.87–1.12)	0.8632	1.50 (1.38–1.64)	<0.0001	1.84 (1.67–2.03)	<0.0001
Heart disease (ref = no)	1.24 (0.96–1.60)	0.1045	1.30 (1.10–1.55)	0.0031	1.68 (1.36–2.10)	<0.0001

Abbreviations: AA refers to African American; IDUD refers to illicit drug use disorder; AUD refers to alcohol use disorder; co-occurrence presents both anxiety and depression; OR = odds ratio; CI = confidence interval.

## Data Availability

The data that support the findings of this study are openly available at https://www.samhsa.gov/data/data-we-collect/nsduh-national-survey-drug-use-and-health (accessed on 25 January 2022) at https://www.samhsa.gov/ (accessed on 25 January 2022).
